# Mitigating the Effects of Oxidative Sperm DNA Damage

**DOI:** 10.3390/antiox9070589

**Published:** 2020-07-06

**Authors:** Taylor Pini, Rachel Makloski, Karen Maruniak, William B. Schoolcraft, Mandy G. Katz-Jaffe

**Affiliations:** Colorado Center for Reproductive Medicine, Lone Tree, CO 80124, USA; drtaylorpini@gmail.com (T.P.); RMakloski@ColoCRM.com (R.M.); KMaruniak@FLColo.com (K.M.); billsgrants@colocrm.com (W.B.S.)

**Keywords:** antioxidant, acai, polyphenol, sperm, DNA damage

## Abstract

Sperm DNA damage is correlated with reduced embryo development and increased miscarriage risk, reducing successful conception. Given its links with oxidative stress, antioxidants have been investigated as a potential treatment, yet results are conflicting. Importantly, individual antioxidants are not identical in composition, and some compounds may be more effective than others. We investigated the use of the polyphenol-rich, high-antioxidant-capacity fruit acai as a treatment for elevated sperm DNA fragmentation (>16%), measured by terminal deoxynucleotidyl transferase dUTP nick end labelling (TUNEL). Following ≥ 74 days of treatment, we observed a significant decrease in sperm DNA fragmentation (−17.0% ± 2.5%) to 11.9 ± 1.7% (0–37%), with a 68.6% success rate (defined as post-treatment TUNEL < 16%). Post-treatment decreases in DNA fragmentation and success rates were not significantly impacted by low motility and/or concentration, or exceptionally high (> 25%) TUNEL. Treatment significantly reduced concentration in men with normal semen parameters, but 88% remained normal. Overall, successful treatment was not associated with age, semen parameters or TUNEL result at baseline. However, body mass index was significantly higher in nonresponders at baseline. This study provides evidence of a low-cost, effective treatment for elevated sperm DNA damage using acai.

## 1. Introduction

An elevated level of sperm DNA damage is a clinical issue in human reproductive medicine due to its associations with reduced fertilization rates [1,2,3], poor embryo development [2,4,5,6,7,8,9] and pregnancy loss [10,11,12,13,14,15]. Because sperm DNA damage may act as a significant barrier to successful conception, an effective treatment is wanting. Given the myriad of evidence that sperm DNA damage and oxidative stress are robustly linked [16,17], antioxidants appear to be a logical treatment option. However, strong evidence for successful treatment of elevated sperm DNA damage with an exogenous antioxidant is lacking. 

A considerable number of studies have investigated how treatment with an antioxidant cocktail, often based on vitamins C and E, impacts sperm DNA fragmentation [18,19,20,21,22,23,24,25,26,27]. However, almost all of these studies were performed on a patient cohort which was not selected based on elevated DNA fragmentation. In addition, many include a mix of men with both normal and abnormal semen parameters, with no compartmentalized analysis to determine the impact of these factors on treatment outcome. Further, while the vast majority of studies show a significant reduction in DNA fragmentation with antioxidant treatment, very few quantify the success rate of treatment (i.e., reduction to a level considered clinically normal) to determine efficacy. On this basis, we suggest there is further need for studies evaluating the effect of antioxidant-based treatments for sperm DNA fragmentation.

A recent large randomized controlled trial (RCT) using an antioxidant cocktail including vitamins C and E, selenium, zinc, folic acid, lycopene and L-carnitine failed to show any improvement in sperm DNA fragmentation following 3 months of treatment [25]. The authors concluded that while reactive oxygen species may be decreased by antioxidant treatment, DNA damage was unaffected. Antioxidant compounds are all unique—chemical constitution, water/lipid solubility and bioavailability can vary significantly between different species. In this context, we hypothesize that antioxidant species other than those used by Steiner, et al. [25] may be able to effectively alleviate sperm DNA damage. The fruit of the palm *Euterpe oleracea*, commonly known as acai, has gained attention in the last two decades for its potential health benefits. Acai is rich in polyphenols, particularly flavonoids and the flavonoid subclass anthocyanins [28,29], and typically displays excellent in vitro antioxidant capacity [30]. Consumption of acai has been shown to decrease plasma oxidant capacity [31], serum oxLDL and malondialdehyde [32] in humans, indicating potential alleviation of oxidative stress in vivo. In rodent models, treatment with acai has significantly reduced tissue levels of malondialdehyde [33] and proteins with oxidative modifications [34], improved germ cell viability and testicular antioxidant enzyme activity following cadmium toxicity [35], and was able to prevent DNA damage in peripheral blood cells in response to a genotoxic agent [36]. These studies suggest that acai could potentially reduce both oxidative stress and DNA damage in vivo. 

Testicular sperm retrieval coupled with intracytoplasmic sperm injection (ICSI) has been suggested as a clinical solution to elevated DNA fragmentation [37]. However, given that this is an invasive and costly procedure, there is a substantial need to investigate alternative treatment strategies, which at the very least can act as a first port of call prior to surgical intervention. Given the previous evidence of its in vivo effects, we employed an acai product with high antioxidant activity to determine its impact on sperm DNA damage in humans. We specifically investigated the impact of acai in a patient cohort selected for elevated DNA fragmentation, assessing both the relative decline in fragmentation and the success rate of treatment (post-treatment DNA fragmentation result below a clinically relevant cut-off). Further, we incorporated subanalyses in order to determine if there is a subset of patients for which this treatment is more or less effective.

## 2. Materials and Methods 

### 2.1. Ethical Approval and Study Design

Patients presenting at the Colorado Center for Reproductive Medicine (Lone Tree, CO, USA) for infertility treatment were recruited by written consent under institutional review board approval (Western Institutional Review Board, Puyallup, WA, approval number 20170532) to a registered clinical trial (ClinicalTrials.gov, NCT03646825). Patients (*n* = 35) were recruited from 2015 to 2019 on the basis of high DNA fragmentation (TUNEL result ≥ 16%). Patients were not excluded on the basis of varicocele, or prior hernia or varicocele repair surgeries. Patients were excluded from the study if surgical sperm retrieval was required, the ejaculate did not contain enough spermatozoa to perform ICSI (<5 × 10^6^ spermatozoa/mL), or there was a history of vasectomy reversal. Semen parameters (including concentration and total motility) and sperm DNA fragmentation (assessed by TUNEL) were measured during an infertility patient workup prior to beginning treatment, and following treatment with 1800 mg freeze-dried acai pulp per day. The dosage regimen was determined based on varied published research with acai (*Euterpe oleracea*) berry pulp, reviewed by Schauss [38]. Patients were treated for a minimum of 74 days to ensure that the treatment period captured at least one new generation of spermatozoa, given that spermatogenesis takes approximately 74 days in humans [39]. Routine clinical data including age, body mass index (BMI), smoking status and days of abstinence were recorded for assessment.

### 2.2. Acai Preparation

Acai was sourced from a commercial manufacturer. Pure acai (*Euterpe oleracea*) berry pulp underwent nonthermal dehydration (freeze-drying) and packaging into vegetable based cellulose capsules (Ecofruits International Inc., South Jordan, UT, USA). Chemical analysis of the lot by the manufacturer reported a total polyphenol content of 6618 mg Gallic acid equivalent (GAE)/100 g, oxygen radical absorbance capacity (ORAC) of 208,628 µmol Trolox equivalent (TE)/100 g and negligible microbial contamination. 

### 2.3. Analysis of Semen Parameters and DNA Fragmentation

Ejaculates were evaluated for semen parameters as part of routine clinical evaluation. Briefly, semen samples were collected by masturbation and allowed to liquefy. Concentration was determined using a Neubauer haemocytometer (Millipore Sigma, Burlington, MA, USA). Total motility was determined by microscopic examination of liquefied, undiluted semen, evaluating a minimum of 200 cells. 

DNA fragmentation was assessed using the terminal deoxynucleotidyl transferase dUTP nick end labelling (TUNEL) assay [40,41]. Ejaculated spermatozoa were washed on a 90%/45% SpermGrad density gradient (Vitrolife), smeared on a slide and fixed in 4% (*v*/*v*) paraformaldehyde. Spermatozoa were permeabilized with 0.1% (*v*/*v*) Triton X-100, blocked (3% (*w*/*v*) bovine serum albumin in phosphate buffered saline, incubated with TUNEL reagent (In situ cell death detection kit, Roche, Indianapolis, IN, USA) and counterstained with 4′,6-diamidino-2-phenylindole (DAPI) for nuclei visualization. Samples were evaluated by fluorescent microscopy, evaluating a minimum of 500 spermatozoa. Both negative (no TUNEL reagent) and positive (spermatozoa treated with DNase) control slides were included to confirm assay performance. Results are reported as the percentage of TUNEL-positive, DNA-damaged spermatozoa. A cut-off of 16% was implemented based on previous studies demonstrating a 14.2% drop in fertilization rates [41], and a four-fold increase in both arrested embryo development and miscarriage risk [42] when TUNEL results were above 15%. Because this cut-off is based on outcome data specific to the TUNEL assay, it is different to cut-offs applied to other DNA damage assays (e.g., sperm chromatin structure assay, 27% [43]). Despite using different cut-offs, results from TUNEL and other DNA damage assays tend to be well-correlated [44,45].

### 2.4. Statistical Analysis

Statistical analysis was carried out in R (v 3.6.3). Data were checked for normality using a Shapiro–Wilk test and homogeneity of variances using an *F*-test. Paired before/after data were analysed using a paired Student’s *t*-test or a Wilcoxon signed rank test (non-normal distribution of differences). To determine the impact of baseline parameters on efficacy of treatment, baseline parameters and the difference between before/after values for each parameter were compared by Student’s *t*-test (Welch correction for unequal variances if required), Wilcoxon rank sum test (non-normal distribution) or Kolmogorov–Smirnov test (non-normal distribution, unequal variances). Proportions of success (post-treatment TUNEL result <16%) were compared between groups by a two sample z-test for equality of proportions. In all cases, tests were two-tailed and employed an α of 0.05. Results are reported as mean ± standard error of the mean (SEM).

## 3. Results

### 3.1. Summary Statistics

Summary data for all patients is presented in Table 1. The cohort included a mix of patients with sperm concentration and motility above and below the World Health Organization (WHO) lower reference limits [46] (Table 2), thus a subanalysis was performed according to these cut-offs. All patients had a baseline TUNEL result above the threshold (≥ 16%), however there was a large spread of results (16–52%). On this basis, a subanalysis comparing moderate (16–25%) to high (>25%) baseline TUNEL was performed. Overall, treatment appeared to be well tolerated, with no gastrointestinal complaints.

### 3.2. Effect of Acai Supplementation on Semen Parameters and DNA Fragmentation

**Figure 1:**
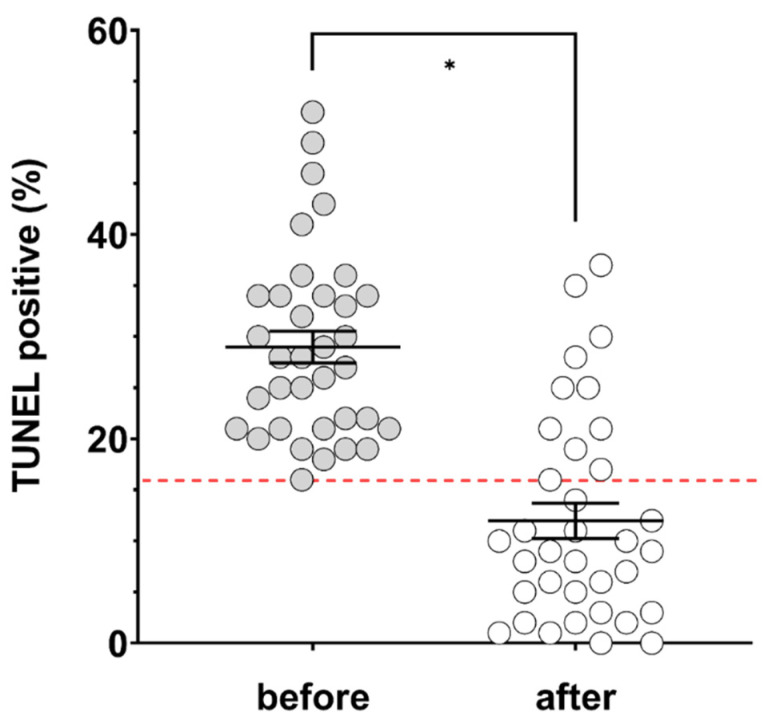
Percentage of terminal deoxynucleotidyl transferase dUTP nick end labelling (TUNEL)-positive spermatozoa before (grey circles) and after (open circles) ≥ 74 days of treatment with acai (*n* = 35). Solid black lines indicate mean and SEM. Red dashed line indicates the clinically relevant cut-off of 16%. * significantly different, *p* value = 0.00000008.

Evaluating the entire cohort, acai treatment did not significantly impact sperm concentration, but did significantly improve motility (Table 2). Before treatment, 37.1% of men had a concentration below the lower reference limit (15 × 10^6^ spermatozoa/mL) and this did not change following treatment. In contrast, while 42.9% of men had total motility below the lower reference limit (40%) at baseline, this decreased to 34.3% of men following treatment. Treatment with acai significantly lowered DNA fragmentation, with a mean post-treatment TUNEL result of 11.97 ± 1.73% and treatment difference of −17.03 ± 2.51% (Figure 1, *p* = 0.00000008). Overall, the success rate for treatment (based on TUNEL result < 16% post-treatment) was 68.6% in the entire cohort. Comparing responders (*n* = 24) to nonresponders (*n* = 11), we observed no significant differences in age, sperm concentration, motility, days of abstinence or TUNEL result at baseline (Table 3). While actual treatment length varied from 75 to 185 days overall (Table 1), there was no significant difference in treatment length between responders and nonresponders (Table 3). Analysing only responders by Pearson correlation, there were no significant correlations between treatment difference for TUNEL result (*r* = 0.07, *p* = 0.73) or motility (*r* = −0.09, *p* = 0.68) and treatment length. Responders had significantly lower BMI at baseline (24.96 ± 0.67 kg/m^2^) compared to nonresponders (29.23 ± 0.93 kg/m^2^, *p* = 0.0008).

### 3.3. Impact of Baseline Semen Parameters on Treatment Outcome

Treatment outcomes for patients with baseline results above (‘normal’, *n* = 17) and below (‘low’, *n* = 18) WHO lower reference limits [46] for concentration (15 × 10^6^ spermatozoa/mL) and/or total motility (40%) were compared. At baseline, the low group showed significantly lower sperm concentration (21.58 ± 10.51 × 10^6^/mL vs 79.59 ± 15.21 × 10^6^/mL, *p* = 0.00006) and motility (26.89 ± 2.82% vs 48.88 ± 1.85%, *p* = 0.0000003), and significantly higher TUNEL results (32.50 ± 1.97% vs 25.29 ± 2.13%, *p* = 0.005) compared to the normal group. Treatment with acai significantly decreased concentration in the normal group (Table 4), though this remained above 15 × 10^6^ spermatozoa/mL for 88.2% (15/17) of patients, and the largest decreases in concentration were observed in men with baseline concentration >100 × 10^6^ spermatozoa/mL. Acai significantly increased motility in the low group, but had no significant effect on concentration (Table 4). While 77.8% (14/18) of men in the low group had a concentration below 15 × 10^6^ spermatozoa/mL at baseline, this improved to 61.1% (11/18) following treatment.

**Figure 2:**
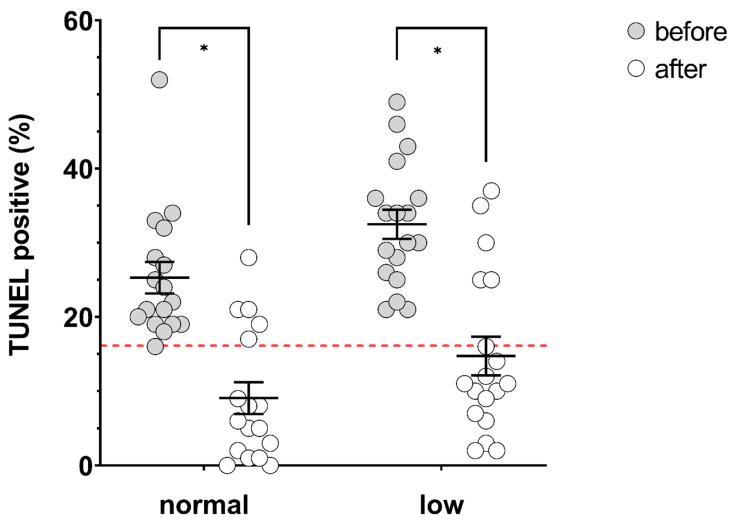
Percentage of TUNEL-positive spermatozoa before (grey circles) and after (open circles) ≥ 74 days of treatment with acai in men with normal (*n* = 17) or low (*n* = 18) baseline semen parameters (‘low’ determined as concentration and/or total motility below WHO lower reference limits (concentration 15 × 10^6^ spermatozoa/mL, total motility 40%). Solid black lines indicate mean and SEM. Red dashed line indicates clinically relevant cut-off of 16%. * significantly different, *p* values = normal; 0.0002, low; 0.0002.

Treatment with acai significantly lowered DNA fragmentation in both groups, with mean post-treatment TUNEL results of 9.06 ± 2.13% (normal, *p* = 0.0002) and 14.72 ± 2.60% (low, *p* = 0.0002) (Figure 2). Treatment differences were not significantly different based on group (normal −16.24 ± 3.37% vs. low −17.78 ± 3.79%, *p* = 0.764). The success rate for treatment (based on TUNEL result < 16% post-treatment) was not significantly different based on normal (70.0%) or low (66.7%) baseline semen parameters (*p* = 1). 

### 3.4. Impact of Baseline DNA Fragmentation on Treatment Outcome

**Figure 3:**
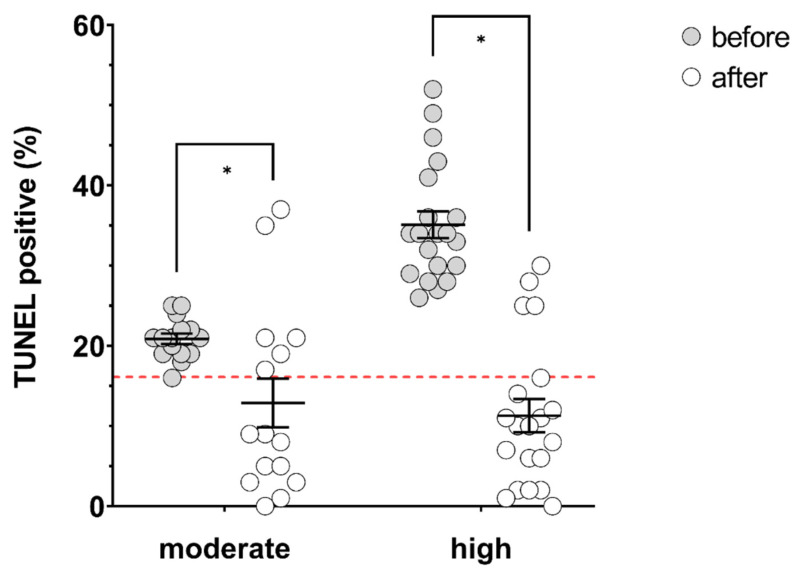
Percentage of TUNEL-positive spermatozoa before (grey circles) and after (open circles) ≥ 74 days of treatment with acai in men with moderate (16 – 25%, *n* = 15) or high (> 25%, *n* = 20) baseline TUNEL results. Solid black lines indicate mean and SEM. Red dashed line indicates clinically relevant cut-off of 16%. * significantly different, *p* values = moderate; 0.02, high; 0.0000002.

Treatment outcomes for patients with moderate (16–25%, *n* = 15) or high (>25%, *n* = 20) baseline TUNEL results were compared. At baseline, the moderate group had significantly lower mean TUNEL results than the high group (20.87 ± 0.65% vs. 35.10 ± 1.67%, *p* = 0.00000007). Treatment with acai had no significant impact on motility or concentration in either group (Table 5). Treatment with acai significantly lowered DNA fragmentation in both groups, with mean post-treatment TUNEL results of 12.87 ± 3.04% (moderate, *p* = 0.02) and 11.30 ± 2.06% (high, *p* = 0.0000002) (Figure 3). Treatment differences were significantly larger in the high group (-23.80 ± 3.02%) compared to the moderate group (−8.00 ± 3.00%, *p* = 0.0009). The success rate for treatment (based on TUNEL result < 16% post-treatment) was not significantly different based on moderate (60.0%) or high (75.0%) baseline TUNEL results (*p* = 0.563).

## 4. Discussion

In this study, we found that consumption of an acai supplement with high antioxidant capacity for a single spermatogenic cycle (74 days) was able to significantly reduce sperm DNA damage measured by TUNEL, with a success rate of 68.6% based on a post-treatment TUNEL result of <16%. Further, we also determined that in this cohort, treatment success was not dependent on baseline concentration or motility being above WHO lower reference limits, nor the initial level of DNA fragmentation, similarly to Greco, et al. [20]. Notably, we found that BMI at baseline was the only significantly different parameter between responders and nonresponders, suggesting that treatment may be less effective in overweight men. This may be due to the chronic inflammatory state produced by interactions between the immune system and excessive adipose tissue [47]. This inflammation contributes to heightened oxidative stress [48], which has been directly observed in the testes of overweight males [49,50]. While acai appears to be effective in treating sperm DNA fragmentation in most men, the excessive oxidative stress associated with overweight and obesity may represent the upper limit of effectiveness for this antioxidant. Due to the small number of nonresponders, further studies looking specifically at the use of acai to treat DNA fragmentation in overweight men are needed to confirm the impact of body mass index on treatment success.

While there was some benefit of treatment to total motility, this was restricted to patients with low motility and/or concentration at baseline. This may be explained by antioxidant rescue of oxidative stress induced immobility [51] in men with abnormal semen parameters. Conversely, we saw a decrease in concentration following treatment in patients with normal semen parameters at baseline. However, concentration remained above the lower reference limit in the majority (88.2%) of patients and the largest declines in concentration were observed in men with very high normal baseline sperm concentrations (>100 × 10^6^ spermatozoa/mL). While mean concentration also decreased following treatment in men with low baseline semen parameters, the difference was not significant. In both groups, the difference in concentration following treatment varied greatly between individuals (−131 to + 132 × 10^6^ overall), making it difficult to draw a meaningful conclusion on the effect of acai on sperm concentration, if any. Further studies employing acai as a treatment for sperm DNA damage should carefully monitor concentration to determine any possible negative effects. As for any treatment strategy, the risks and benefits must be weighed for individual patients and in this case, reducing DNA damage from high to acceptable levels may be found to offer more significant benefit than any potential decline in normal sperm concentration.

This is the first published study to investigate the impact of acai on sperm DNA damage. While there are no studies to which we can directly compare our results, we can look to the many other studies employing antioxidant-based treatments for comparison. In most studies, employing an antioxidant cocktail (typically containing vitamins C and E, as well as zinc, selenium, l-carnitine and coQ10), or even individual antioxidants such as *N*-acetyl-cysteine, treatment for 2–3 months significantly reduced sperm DNA fragmentation measured by sperm chromatin structure assay (SCSA), sperm chromatin dispersion (SCD) or TUNEL [18,19,20,21,22,24,27,52,53]. In most cases, the average decrease following treatment was modest (<10%) [18,22,24,27,52], despite being statistically significant. Even in those studies with larger treatment differences [19,21], the post-treatment decrease in DNA fragmentation was lower than in the current study (−17%). A recent large RCT employing an antioxidant cocktail did not show any significant improvement in sperm DNA fragmentation following treatment [25]. The authors highlighted sample size as a limitation, and indeed only 19 men diagnosed with high DNA fragmentation received antioxidant treatment. The extent of treatment effect we observed in the current study may be due to selection of a larger patient cohort on the basis of high DNA fragmentation, as well as the unique chemical composition of acai compared to previously tested antioxidant cocktails.

A broad range of antioxidants have been employed in previous studies, however it is important to note that each antioxidant species is unique in its chemical composition and biological impact. Acai is particularly rich in polyphenols, especially flavonoids and anthocyanins [28,29]. Information on the tissue bioavailability and in vivo antioxidant capacity of polyphenols is limited or nonexistent, however a number of studies suggest important effects on oxidative stress and DNA damage in vivo. Studies across humans, animals and cell models provide evidence that polyphenols may increase the activity and expression of endogenous antioxidant enzymes, and reduce both markers of oxidative DNA damage and occurrence of strand breaks (often in response to DNA-damaging agents) [54,55]. Polyphenols have further been shown to increase the expression of DNA damage repair genes in yeast [56] and mice [57] under UV challenge. These studies suggest that polyphenols are biologically active when consumed, however the exact mechanism by which they may limit DNA damage in spermatozoa is unknown. 

Research into the impact of polyphenols on male reproduction is scarce; a study in sheep has shown that under restraint stress, a polyphenol-rich dietary supplement improved semen parameters and the expression of antioxidant enzymes in the testes [58]. A recent study showed that administration of an oil extract from *E. oleracea* fruits was able to limit the damaging effects of cadmium toxicity, rescuing germ cell viability, restoring testosterone concentration and significantly increasing superoxide dismutase (SOD) activity in the testis [35]. These results suggest that acai contains compounds which actively contribute to the restoration of homeostasis in tissues under considerable oxidative challenge. One in vitro study suggested that some individual polyphenols are not appropriate additives to sperm handling media due to cytotoxicity [59]. However, the relevance of this study to the in vivo environment is limited, given the inherent metabolic processing of polyphenols and likely low tissue concentrations following consumption. Further, polyphenols have been shown to be a beneficial medium component for sperm function in several other species, including pigs [60], sea urchins [61] and cattle [62]. Generally, while there is some evidence for possible mechanisms by which acai may impact sperm DNA damage, further molecular studies are needed to determine how this effect is mediated.

While our results are promising, they are tempered by the limitations of this study. This was a relatively small patient cohort, and subanalyses involved groups which were smaller still. Given the success we observed, we believe our results call for further investigation into acai as a therapeutic treatment for high sperm DNA fragmentation. Because of limited access to sample amount, we were only able to assess concentration, motility and DNA damage in this study; future studies should endeavour to explore a wider array of outcomes, particularly those relevant to oxidative stress (e.g., malondialdehyde and 4-hydroxynonenal levels, total antioxidant capacity and activity of individual antioxidant enzymes). Due to our limited numbers, we were also unable to perform a robust analysis on the impact of treatment on reproductive outcomes such as fertilization rate, embryo development and pregnancy loss. Further, while we observed a beneficial effect, it is unknown how long the benefit would persist following cessation of treatment with acai. These are important outcomes to consider, and we hope to address these with further studies.

## 5. Conclusions 

This study provides evidence for the effectiveness of polyphenol-rich, high-antioxidant-potential acai in treating sperm DNA fragmentation. This treatment had a good overall success rate (68.6%), and did not appear to be any less effective in men with abnormal semen parameters or very high (>25%) DNA fragmentation at baseline. BMI may play a role in the success of treatment, but this requires further substantiating evidence. In men with normal sperm parameters, acai treatment may decrease concentration, however this needs to be confirmed in a larger cohort, and the risk and benefit of this treatment approach weighed for individual patients. While further research is needed, this is a promising first step towards the use of a novel plant-derived antioxidant as a therapeutic treatment for elevated sperm DNA damage.

## Figures and Tables

**Table 1:** Summary data for all patients.

Parameter	Mean ± SEM	Range	*n*
Age (years)	39.51 ± 6.68	33–59	35
Baseline TUNEL (%)	29.00 ± 4.90	16–52	35
Baseline abstinence (days)	3.71 ± 0.64	0–10	34
Post-treatment abstinence (days)	3.33 ± 0.64	1–10	27
Body mass index (kg/m^2^)	26.34 ± 4.52	18.6–34.2	34
Treatment length (days)	109.51 ± 18.51	75–185	35
Smoker (%)	5.71 (2/35)	-	35

**Table 2:** Semen parameters at baseline and following treatment with acai in all patients (*n* = 35).

Parameter	Before	After	Treatment Difference	*p* Value *
Concentration (×10^6^/mL)	49.75 ± 10.30 (0.2–224)	33.87 ± 6.01 (1–162)	−15.88 ± 2.56 (−131–132)	0.083
Total motility (%)	37.57 ± 2.53 (9–70)	45.74 ± 3.56 (2–83)	8.17 ± 3.37 (−40–45)	0.021

* *p* value of paired *t*-test comparing before/after results. Data are presented as mean ± SEM (range).

**Table 3:** Parameters in responders (TUNEL result < 16% post-treatment) and nonresponders (TUNEL result ≥ 16% post-treatment).

Parameter	Responders (*n* = 24)	Non-Responders (*n* = 11)	*p* Value *
Age (years)	39.33 ± 1.15 (33 – 59)	39.91 ± 1.28 (33 – 47)	0.422
Baseline concentration (×10^6^/mL)	54.44 ± 13.71 (0.2 – 224)	39.53 ± 13.74 (1.2 – 127)	0.696
Baseline total motility (%)	39.21 ± 3.02 (9 – 70)	34.00 ± 4.62 (13 – 52)	0.346
Baseline TUNEL (%)	30.54 ± 2.07 (16 – 52)	25.64 ± 1.75 (19 – 36)	0.233
Baseline body mass index (kg/m^2^)	24.96 ± 0.67 (18.6 – 32.5)	29.23 ± 0.93 (23.7 – 34.2)	0.0008
Baseline abstinence (days)	3.74 ± 0.35 (1 – 10)	3.64 ± 0.58 (0 – 8)	0.892
Post-treatment abstinence (days)	3.30 ± 0.42 (1 – 10)	3.43 ± 0.61 (1 – 5)	0.607
Treatment length (days)	107.58 ± 4.62 (75 – 161)	113.73 ± 8.26 (90 – 185)	0.434

* *p* value of *t*-test comparing parameters between responders and nonresponders. Data are presented as mean ± SEM (range).

**Table 4:** Semen parameters at baseline and following treatment with acai in men with normal or low sperm concentration and/or motility at baseline ^a^.

Parameter	Normal Concentration/Motility (*n* = 17)				Low Concentration/Motility (*n* = 18)			
	Before	After	Treatment Difference	*p* Value *	Before	After	Treatment Difference	*p* Value *
Concentration (×10^6^/mL)	79.59 ± 15.2 (18–224)	51.68 ± 9.97 (5.5–162)	−27.91 ± 14.03 (−113.5–132)	0.008	21.58 ± 10.51 (0.2–190)	17.06 ± 4.21 (1–59)	−4.52 ± 8.44 (−131–30.5)	0.196
Total motility (%)	48.88 ± 1.85 (40–70)	51.00 ± 4.78 (5–76)	2.12 ± 4.70 (−40 – 26)	0.658	26.89 ± 2.82 (9–51)	40.78 ± 5.11 (2–83)	13.89 ± 4.54 (−18–45)	0.007

^a^ ‘Low’ determined as either concentration and/or total motility below WHO lower reference limits (concentration 15 × 10^6^ spermatozoa/mL, total motility 40%). * *p* value of paired *t*-test comparing before/after results within normal or low group. Data are presented as mean ± SEM (range).

**Table 5:** Semen parameters at baseline and following treatment with acai in men with moderate (16–25%) or high (>25%) TUNEL at baseline. * *p* value of paired *t*-test comparing before/after results within moderate or high group.Data are presented as mean ± SEM (range).

Parameter	Moderate TUNEL Result (*n* = 15)				High TUNEL Result (*n* = 20)			
	Before	After	Treatment Difference	*p* Value *	Before	After	Treatment Difference	*p* Value *
Concentration (×10^6^/mL)	74.80 ± 19.19 (4.5–224)	45.50 ± 11.23 (3.5–162)	−29.40 ± 17.48 (−131–132)	0.115	30.97 ± 9.22 (0.2–178)	25.23 ± 5.84 (1–107)	−5.75 ± 5.40 (−71.5–30.5)	0.601
Total motility (%)	45.07 ± 3.53 (13–70)	51.67 ± 5.66 (2–83)	6.60 ± 5.04 (−21–34)	0.211	31.95 ± 3.03 (9–53)	41.30 ± 4.43 (5–75)	9.35 ± 4.63 (−40–45)	0.058
